# Effects of polymorphisms in the *MTHFR* gene on 5-FU hematological toxicity and efficacy in Thai colorectal cancer patients

**DOI:** 10.3389/fonc.2022.916650

**Published:** 2022-07-15

**Authors:** Chalirmporn Atasilp, Rinradee Lenavat, Natchaya Vanwong, Phichai Chansriwong, Ekaphop Sirachainan, Thanyanan Reungwetwattana, Pimonpan Jinda, Somthawin Aiempradit, Suwannee Sirilerttrakul, Monpat Chamnanphon, Apichaya Puangpetch, Nipaporn Sankuntaw, Patompong Satapornpong, Chonlaphat Sukasem

**Affiliations:** ^1^ Chulabhorn International College of Medicine, Thammasat University, Pathum Thani, Thailand; ^2^ Department of Clinical Chemistry, Faculty of Allied Health Sciences, Chulalongkorn University, Bangkok, Thailand; ^3^ Division of Medical Oncology, Department of Medicine, Faculty of Medicine Ramathibodi Hospital, Mahidol University, Bangkok, Thailand; ^4^ Division of Pharmacogenomics and Personalized Medicine, Department of Pathology, Faculty of Medicine Ramathibodi Hospital, Mahidol University, Bangkok, Thailand; ^5^ Laboratory for Pharmacogenomics, Clinical Pathology, Somdetch Phra Debharatana Medical Centre, Ramathibodi Hospital, Bangkok, Thailand; ^6^ Department of Pathology, Faculty of Medicine, Srinakharinwirot University, Nakhonnayok, Thailand; ^7^ Division of General Pharmacy Practice, Department of Pharmaceutical Care, College of Pharmacy, Rangsit University, Pathum Thani, Thailand; ^8^ Excellence Pharmacogenomics and Precision Medicine Centre, College of Pharmacy, Rangsit University, Pathum Thani, Thailand

**Keywords:** 5-fluorouracil, colorectal cancer, *MTHFR* polymorphisms, toxicity, efficacy

## Abstract

**Background:**

The two common methylenetetrahydrofolate reductase (*MTHFR*) polymorphisms 677G>A and 1298A>C may have been affecting 5-FU toxicity in cancer patients for decades. Drug efficacy has also been shown by previous studies to be affected. In this study, we investigated the effects of these polymorphisms on 5-FU hematological toxicity and treatment efficacy, to provide enhanced pharmacological treatment for cancer patients.

**Methods:**

This is a retrospective study involving 52 Thai colorectal cancer patients who were treated with 5-FU based therapy, using TaqMAN real-time PCR to genotype the *MTHFR* polymorphisms (677G>A and 1298A>C). The toxicity and response rate were assessed using standardized measures.

**Results:**

Neutropenia was significantly more likely to be experienced (*P*=0.049, OR=7.286, 95% CI=0.697-76.181) by patients with the *MTHFR* 677G>A polymorphism, in the same way as leukopenia (*P* =0.036, OR=3.333, 95%CI=2.183-5.090) and thrombocytopenia (*P*<0.001, OR=3.917, 95%CI=2.404-6.382). The *MTHFR* 1298A>C polymorphism had no statistical association with hematological toxicity in 5-FU treatment. The response rate to 5-FU was not significantly affected by these two polymorphisms.

**Conclusion:**

The *MTHFR* polymorphism 677G>A is a significant risk factor for developing leukopenia, neutropenia and thrombocytopenia as toxic effects of 5-FU therapy in cancer patients. Therefore, patients receiving 5-FU-based therapy should be aware of their polymorphisms as one risk factor for experiencing severe toxicity.

## Introduction

Data from GLOBOCAN 2018 reveals that colorectal cancer is the third most deadly and fourth most commonly diagnosed cancer in the world (1). When restricted to Thailand, a 2021 study shows that colorectal cancer is also the third most common cancer, contributing to 11% of the total national cancer burden (2).

The cell-cycle specific anti-metabolite 5-fluorouracil (5-FU) is one of the most commonly used drug regimens for the treatment of many cancers including colorectal cancer. It is a pyrimidine analog which acts to interfere with DNA synthesis. However, 69% of patients undergoing 5-FU therapy for colon cancer experience neutropenia (3). Therefore, for certain patients, this serious toxicity is the main limitation to its use, as increased susceptibility to infections can occur which is potentially life-threatening. The enzyme methylenetetrahydrofolate reductase (MTHFR) plays a key role in the metabolism of 5-FU. Fluorouracil is irreversibly reduced by MTHFR enzyme to the compound 5-methyltetrahydrofolate (5-MTHF), which is later used in DNA methylation through the conversion of homocysteine to methionine (4). This step normally involves the conversion of dUMP to dTMP by thymidylate synthase. As a result, DNA synthesis is directly disrupted, leading to cell damage and apoptosis. A decrease in *MTHFR* has been linked to an increase in 5,10-MTHF the substrate for MTHFR and thus an increase in 5-FU toxicity (5).

Genetics play an important role in individual differences among patients that have shown different levels of pharmacological toxicity. The *MTHFR* gene is located on chromosome 1p36.22 and is prone to polymorphisms. The two most common polymorphisms studied are G677A (alanine to valine) and A1298C (glutamine to alanine). Several studies have shown the effects of these two polymorphisms of the *MTHFR* gene in reduced enzymatic activity in the metabolism of 5-FU (6, 7). For the G677A polymorphism, homozygous TT individuals have 30% of expected enzyme activity, whilst heterozygous CT individuals have 65% of such activity, in comparison to the most common genotype CC (8).

The G677A single nucleotide polymorphism (SNP-rs1801133) of the *MTHFR* gene is most commonly linked with hematologic toxicity, and this correlation has been shown in the Chinese population (p = 0.005) (9). A previous study found that the 677 GG genotype is associated with toxicity (odds ratio = 1.83, *P* = 0.01) (10). On the other hand, another study concluded that the G677A genotype did not significantly affect the cytotoxic activity of 5-FU (11). As for the 1298A>C genotype, a study found it was linked to toxicity (4).

The variant 1298 A>C (rs1801131) has been demonstrated to increase 5-FU efficacy in patients with colorectal cancer, by increasing progression-free survival (PFS - time from operation to death or censorship), whereas 677 G>A showed no correlation (6, 11). However, in another study, the *MTHFR* G677A mutation was shown to increase chemosensitivity to 5-FU in colon and breast cancer (7). Another study showed a specific association of a good clinical response to FOLFOX therapy (leucovorin, fluorouracil, and oxaliplatin) with the two *MTHFR* polymorphisms 677G>A and 1298A>C (p=0.040) (12).

Hence, the results from previous studies are currently inconclusive and controversy still exists over whether these two SNPs of the *MTHFR* gene really lead to increased hematological toxicity and treatment efficacy (13, 14). Additionally, few studies have reported the use of 5-FU in Thai colorectal cancer patients.

The aim of this study was to investigate the effects of the specific SNPs of *MTHFR* polymorphism on the association with hematological toxicity as an adverse drug reaction of 5-FU in Thai colorectal cancer patients, along with the efficacy of the drug.

## Methods

### Eligible patients

**Figure 1:**
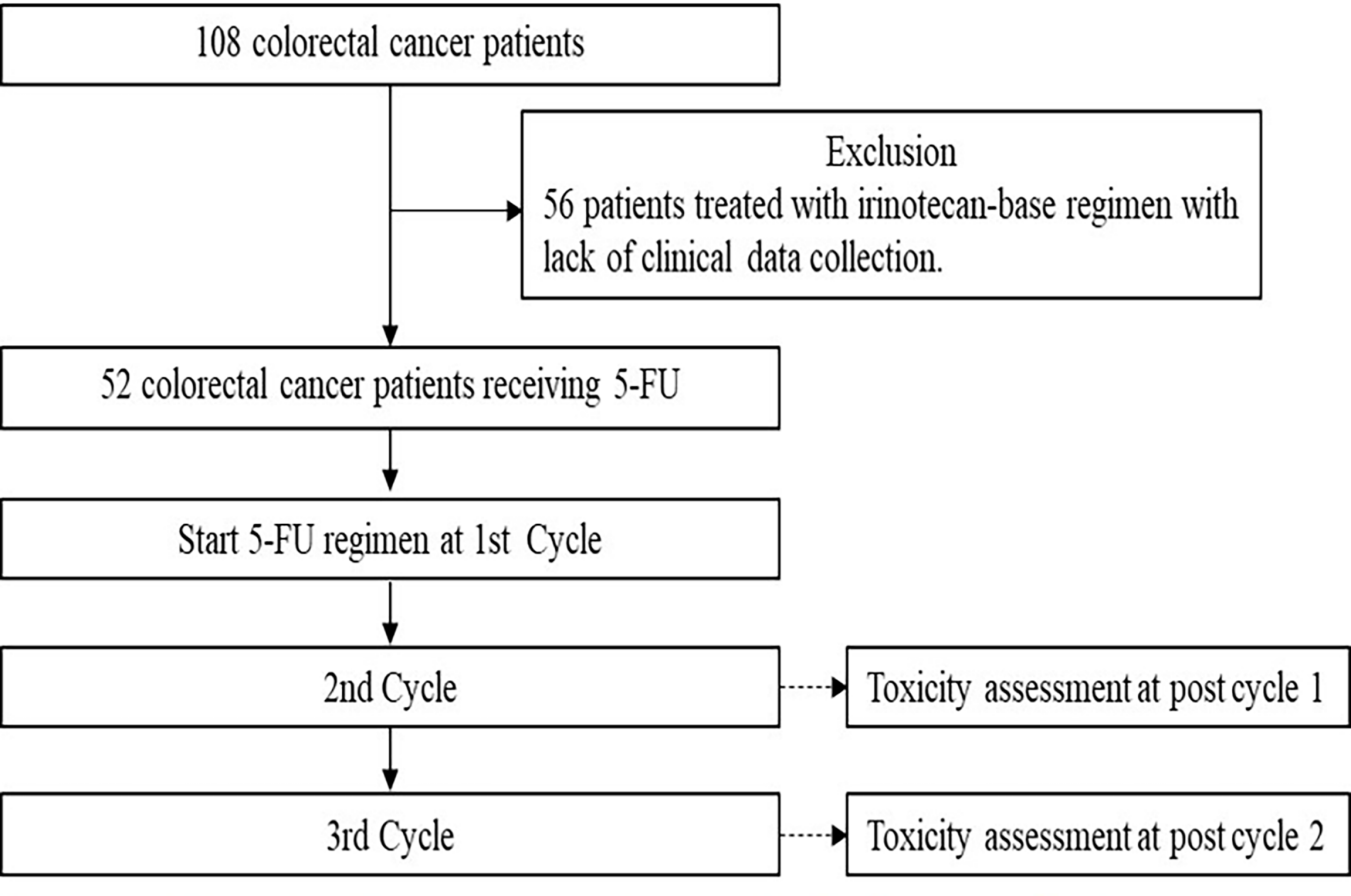
Flow chart for patient screening. A total of 108 metastatic colorectal cancer patients were genotyped for generic polymorphisms and 56 patients who did not treated with 5-flourouracil-based chemotherapy were excluded. Of the 52 patients treated with 5-flourouracil-based chemotherapy were included in this analysis.

A total of 108 colorectal cancer patients were recruited between October 2020 and October 2021 from the Division of Oncology, Department of Medicine, Faculty of Medicine, Ramathibodi Hospital, Mahidol University, Thailand. The clinical eligibility criteria to recruit patients were as follow: histologically or cytologically confirmed to be diagnosed with colorectal cancer, having not received fluorouracil before (first or second cycles of treatment), aged at least 18 years, Eastern Cooperative Oncology Group (ECOG) performance status 0-2, life expectancy > 3 months, neutrophil count ≥ 1.5 × 10^9^/L, platelet count ≥ 8 × 10^10^/L, serum creatinine ≤ 1.25, upper limit normal (ULN), total bilirubin ≤ 1.25 ULN, and alanine aminotransferase and aspartate aminotransferase ≤ 2.5 ULN. Fifty-two patients who had been treated with 5-FU-based-chemotherapy were analyzed for toxicity assessment. The flow chart for patient screening is shown in **Figure 1**. Patients who were excluded from the study had one or more of the following characteristics: liver or kidney disease, pregnancy, or did not consent to the study.

This study was approved by the Ethics Review Committee on Human Research of the Faculty of Medicine Ramathibodi Hospital, Mahidol University, Thailand (MURA2020/1613) and was conducted in accordance with the Declaration of Helsinki. The study procedure was clearly explained to the patients before the study and written consent forms were issued accordingly.

### Genotyping analysis

Peripheral blood was collected in ethylenediaminetetraacetic acid (EDTA) tubes. Firstly, the MagNA pure compact system (Roche, Manheim, Germany) was used to purify the DNA in the blood samples. Following this, nanodrop microvolume technology (Thermo Fisher Scientific, DE, USA) was used to check the purity of the DNA, relying on the surface tension qualities of the sample liquified into a column using a 260/280 ratio. A score of 1.80-2.00 was considered to represent of good purity. The methylenetetrahydrofolate reductase *(MTHFR)* polymorphisms were examined by TaqMAN real-time PCR, which involved amplifying and detecting targeted polymorphisms quantitatively. The two SNPs studied were 677G>A (C_1202883_20) and 1298A>C (C_850486_20).

### Drug administration

As for the drug administration the most common drug or drug combination received was 5-fluorouracil + leucovorin (5-FU at 425 mg/m^2^/day, 5 days + leucovolin 30 mg), which 22 patients (42.3%) received. Another set of 20 patients received FOLFOX (intravenous oxaliplatin 85 mg/m^2^ on day 1 and a 2-hour infusion of leucovorin 200 mg/m^2^ followed by bolus 5-FU 400 mg/m^2^ and a 22-hour continuous infusion of 5-FU 600 mg/m^2^ for two consecutive days with treatment repeated every two weeks) (38.5%) while the following drugs were received by less than 10% of patients: modified FOLFOX (intravenous oxaliplatin 85 mg/m^2^ on day 1 and a 2-hour infusion of leucovorin 200 mg/m^2^ followed by bolus 5-FU 400 mg/m^2^ and a 22-hour continuous infusion of 5-FU 1200 mg/m^2^ for two consecutive days with this treatment repeated every two weeks) and FOLFOX + Avastin (Avastin 5–10 mg/kg intravenous infusion once every 2 weeks; intravenous oxaliplatin 85 mg/m^2^ on day 1 and a 2-hour infusion of leucovorin 200 mg/m^2^ followed by bolus 5-FU 400 mg/m^2^ and a 22-hour continuous infusion of 5-FU 600 mg/m^2^ for two consecutive days with this treatment repeated every two weeks) which 7 patients (13.5%) and 3 patients (5.8%) received, respectively.

### Outcome

Toxicity was assessed according to National Cancer Institute Common Toxicity Criteria for Adverse Events version 5.0 (CTCAE). Grades 1-4 were considered to be toxic. Grades 3–4 were considered severely toxic. The specific toxicity this study focused on was hematological toxicity.

The efficacy or response rate of cancer after drug administration was measured in accordance with the Response Evaluation Criteria in Solid Tumors (RECIST) version 1.0, which comprises the following ratings: complete response (CR), partial response (PR), stable disease (SD) and progressive disease (PD).

### Statistical analysis

Descriptive statistics were used to describe the clinical characteristics of the subjects. Data are reported as medians (interquartile range, IQR). Associations among the genetic polymorphisms (alleles and genotypes), adverse events (toxicity), response rates, and clinical characteristics (age and risk group) were evaluated with the χ2 test, or Fisher’s exact test. The odds ratio (OR) and 95% confidence interval (CI) were calculated from the contingency table. All statistics were calculated using SPSS software version 22 (Chicago, IL, USA), and the statistical significance was set at p < 0.05.

## Result

### Allele and genotype frequency

A total of 108 colorectal cancer patients receiving 5-fluorouracil based therapy were genotyped for two SNPs: *MTHFR* 677G>A and *MTHFR* 1298A>C. The genotype and allele frequencies are shown in **Table 1**. The prevalence of *MTHFR* 677G>A polymorphism is 0.17. Most of the patients (75/108; 69.4%) had the homozygous wild type (GG), while 26.9% (29/108) had the heterozygous variant (GA) and 3.7% (4/108) had the homozygous variant. As for the *MTHFR* 1298A>C polymorphism, the prevalence of the allele was 0.27. Most patients (55/108; 50.9%) had the homozygous wild type (AA), while 43.5% (47/108) had the heterozygous variant (AC) and 5.6% (6/108) had the homozygous variant (CC).

**Table 1:** Allele and genotype frequencies of 108 Thai metastatic colorectal cancer patients.

Gene	Polymorphism	Allele frequency	Genotype frequency (%)
***MTHFR***	677G>A (rs1801133)		
	G	0.83	
	A	0.17	
	GG		75 (69.4)
	GA		29 (26.9)
	AA		4 (3.7)
***MTHFR***	1298A>C (rs1801131)		
	A	0.73	
	C	0.27	
	AA		55 (50.9)
	AC		47 (43.5)
	CC		6 (5.6)

### Patient characteristics

The clinical characteristics of the colorectal cancer patients are summarized in **Table 2**. Of those 108, 52 colorectal cancer patients who were treated with 5-FU-based-chemotherapy were included in the study. A total of 31 were male, 21 were female and the mean age of the sample was 60 years (range 47-73). The majority (69.2%) had an ECOG performance status of 0. The most common site of disease was the rectum (50%). The liver was the most common site of metastasis (47.7%). There were no statistically significant differences between clinical characteristics and hematological toxicity including neutropenia, leucopenia, thrombocytopenia, and anemia (data not shown).

**Table 2:** Patient characteristics. (N=52).

Characteristics	Number of patients (%)
**Age (years), median ± IQR**	60, ± 13
**Gender**	
**Male**	31 (59.6)
**Female**	21 (40.4)
**ECOG performance status**	
**0**	36 (69.2)
**1**	15 (28.8)
**2**	1 (1.92)
**Site of disease**	
**Rectum**	26 (50)
**Sigmoid**	14 (26.9)
**Right side**	4 (7.7)
**Rectosigmoid**	4 (7.7)
**Left side**	2 (3.9)
**Transverse**	2 (3.9)
**Sites of metastases**	
**Liver**	31 (47.7)
**Lung**	16 (24.6)
**Others**	18 (27.7)
**Histopathology type**	
**Well differentiated**	15 (28.8)
**Moderately differentiated**	33 (63.5)
**Poorly differentiated**	4 (7.7)
**Line of treatment**	
**First line**	47 (90.4)
**Second line**	5 (9.6)
**Treatment regimen**	
**5-Fluorouracil + Leucovolin**	22 (42.3)
**FOLFOX**	20 (38.5)
**mFOLFOX**	7 (13.5)
**FOLFOX + Avastin**	3 (5.8)

### Correlation of MTHFR 677G>A and 1298A>C polymorphisms with hematological toxicity in 5-FU treatment

The clinical association is summarized in **Table 3**. As for the first cycle of treatment, the *MTHFR* 677G>A polymorphism was statistically associated (*P*=0.036, OR=3.333, 95%CI = 2.183-5.090) with grade 3-4 leukopenia. For the second cycle, the *MTHFR* 677G>A polymorphism was statistically associated (*P* =0.049, OR=7.286, 95% CI=0.697-76.181) with grade 3-4 neutropenia. The MTHFR 677G>A polymorphism was significantly statistically associated with grade 1-4 thrombocytopenia (*P <*0.001, OR=3.917, 95%CI=2.404-6.382).

**Table 3:** Different grades of toxicities in first and second cycles (N=52) caused by 5-FU-based chemotherapy in the patients with different genotypes of MTHFR polymorphism.

Toxicity	Gene	Genotype	N	First cycle				Second cycle			
				Grade 1-4^a^n (%)	*P*	Grade 3-4^b^n (%)	*P*	Grade 1-4^a^n (%)	*P*	Grade 3-4^b^n (%)	*P*
Anemia	*MTHFR* 677G>A	GG	35	19 (54.3)	0.223	0 (0.0)	ND	14 (40.0)	0.333	0 (0.0)	ND
		GA + AA	17	12 (70.6)		0 (0.0)		9 (52.9)		0 (0.0)	
	*MTHFR* 1298A>C	AA	31	17 (54.8)	0.343	0 (0.0)	ND	13 (41.9)	0.650	0 (0.0)	ND
		AC + CC	21	14 (66.7)		0 (0.0)		10 (47.6)		0 (0.0)	
Leucopenia	*MTHFR* 677G>A	GG	35	8 (22.9)	0.544	0 (0.0)	0.036*	11 (31.3)	0.224	0 (0.0)	ND
		GA + AA	17	5 (29.4)		2 (11.8)		8 (47.1)		0 (0.0)	
	*MTHFR* 1298A>C	AA	31	7 (22.6)	0.579	2 (6.5)	0.568	12 (38.7)	0.660	0 (0.0)	ND
		AC + CC	21	6 (28.6)		0 (0.0)		7 (33.3)		0 (0.0)	
Neutropenia	*MTHFR* 677G>A	GG	35	11 (31.4)	0.768	5 (14.3)	0.712	10 (28.6)	0.945	1 (2.9)	0.049*
		GA + AA	17	4 (23.5)		3 (17.6)		5 (29.4)		3 (17.6)	
	*MTHFR* 1298A>C	AA	31	9 (29.0)	0.968	4 (12.9)	0.488	9 (29.0)	0.968	2 (6.5)	0.640
		AC + CC	21	6 (28.6)		4 (19.0)		6 (28.6)		2 (9.5)	
Thrombocytopenia	*MTHFR* 677G>A	GG	35	0 (0.0)	ND	0 (0.0)	ND	0 (0.0)	<0.001*	0 (0.0)	0.195
		GA + AA	17	0 (0.0)		0 (0.0)		5 (29.4)		1 (5.9)	
	*MTHFR* 1298A>C	AA	31	0 (0.0)	ND	0 (0.0)	ND	5 (16.1)	0.059	1 (3.2)	0.405
		AC + CC	21	0 (0.0)		0 (0.0)		0 (0.0)		0 (0.0)	

ND = Not determined; * P value < 0.05 was considered statistically significant. ^a^Grade 1–4 was considered as toxicity. ^b^Grade 3–4 was considered as severe toxicity.

Simultaneously, the *MTHFR* 677G>A polymorphism had no statistical significance in 5-FU treatment in conjunction with the first and second cycles for all grades of anemia toxicity, the first cycle of grade 1-4 leukopenia, the second cycle for all grades of leukopenia, the first cycle of all grades of neutropenia, or second cycle grade 1-4 neutropenia. For all grades of thrombocytopenia, the first cycle of treatment had no significant association and the second cycle had no association for severe toxicity.

The *MTHFR* 1298A>C polymorphism had no statistical association with either the first or second cycles of 5-FU treatment, or with any grade or types of hematological toxicity. Anemia as an effect of hematological toxicity caused by 5-FU treatment was not statistically associated with the two polymorphisms (*MTHFR* 677G>A and *MTHFR* 1298A>C) in either first or second cycles. There were no statistically significant differences between the combined *MTHFR* polymorphisms and hematological toxicities in the first or second cycles.

### Response rate

There was no statistical significance in the response rate of patients with *MTHFR* 677G>A and *MTHFR* 1298A>C polymorphisms. This clinical data is summarized in **Table 4**. There were no statistically significant differences between response rate and clinical characteristics in the first or second cycles.

**Table 4:** Response rate of Thai colorectal cancer patients (N=52) in 5-FU based therapy.

Gene	Genotype	N	Responders (CR + PR) (N=7)	Non-responders (SD + PD) (N=45)	*P* value
*MTHFR* 677G>A	GG	35	5 (14.3)	30 (85.7)	1.000
	GA + AA	17	2 (11.8)	15 (88.2)	
*MTHFR* 1298A>C	AA	31	5 (16.1)	26 (83.9)	0.721
	AC + CC	21	2 (9.5)	19 (90.5)	

p value < 0.05 was considered as severe toxicity.

## Discussion

To our knowledge, there is not yet another study on the association of *MTHFR* 677G>A and *MTHFR* 1298A>C polymorphisms with 5-FU treatment in Thai colorectal cancer patients. Our findings suggest that the *MTHFR* 677G>A polymorphism is a high-risk factor that contributes to hematologic toxicity associated with 5-FU-based therapy in both first and second cycles of treatment.

The prevalence of *MTHFR* 677A allele frequencies in the Chinese population found in other studies was much higher than that in this study, being 0.56 compared to 0.17, respectively (15). However, similar allele frequencies were found for *MTHFR* 1298C in a minority group of the Chinese population, with 0.26 compared to the 0.27 in this study (16). In comparison to other ethnic groups such as the Caucasian population, both allele frequencies 677A and 1298C for the polymorphisms were higher than those found in this study, with 0.33 and 0.38 respectively (17). Similarly, a study investigating the female Turkish population found both allele frequencies 677A and 1298C to be higher (0.26 and 0.37 respectively) than values found in the Thai population (18).

This present study found that having the *MTHFR* 677A allele increases the risk of severe neutropenia sevenfold as a symptom of hematological toxicity in the second cycle of 5-FU treatment (95% CI =0.697-76.181). This finding is in accordance with another study investigating the Chinese population which found that the 677A allele was closely associated with severe neutropenia (p=0.043) (19). Similarly, a study on Bangladeshi patients found that the 677A allele could predict grade 3 or 4 neutropenia as a result of 5-FU toxicity (9). A meta-analysis consisting mostly of Asians and Europeans also found that gastric cancer patients with the GG or GA genotype tended to experience less severe hematological toxicity than those with the AA genotype[(GG+GA)/TT OR=0.66, 95% CI: 0.48-0.91] (20). These findings are opposed to several studies that found that the 677A allele was not associated with hematological toxicity (11). As a matter of fact, one study actually found the 677GG genotype to be related to toxicity(odds ratio = 1.83, CI = 1.13-2.96, P = 0.01) (10).

In this study, leukopenia is three times (*P*=0.036, OR=3.333, 95%CI = 2.183-5.090) more likely for patients with the *MTHFR* 677G>A polymorphism as a result of toxicity caused by receiving 5-FU. In contrast, Matthias Schwab et al. found no significant association between the *MTHFR* 677G>A polymorphism and all grades of leukopenia (21).

One interesting finding is that patients with the *MTHFR* 677G>A polymorphism have around four times (*P*<0.001, OR=3.917, 95%CI=0.697-76.181) the risk of experiencing thrombocytopenia as a side effect of 5-FU therapy. From Ahmad, F, et al., and Franchini, M., et al., reported that *MTHFR* 677C/T was associated with an increased prothrombotic risk factor (22, 23). This result is in alignment with V. Adamo *et al.* who reported that one third of patients with the homozygous variant genotype (*MTHFR* 677 AA) faced grade 3 thrombocytopenia after the first cycle of treatment (24).

The *MTHFR* 1298A>C polymorphism was discovered to be not statistically associated with any hematological toxicity in this study. Previous studies have reported similar findings, as researchers also did not find any statistical significance correlated with the 1298C allele and 5-FU toxicity (10). This finding is further in agreement with another study on Indian patients, which also did not find any significant association between the 1298C allele and 5-FU toxicity (25). However, another study on the French population reported that 1298CC genotype was associated with toxicity(p = 0.0018), in the opposite manner to the current study (4). Furthermore, V. Adamo et al. found that one third of patients with homozygous wild genotype (MTHFR 1298 AA) experienced grade 3 granulocytopenia and grade 3 thrombocytopenia (24).

As for the response rate, although this study did find better response rates, patients with responders was low number 13.5% (7/52). Therefore, the difference regarding the association with MTHFR 677A and MTHFR 1298C alleles was not statistically significant.

Previous studies have reported similar results regarding drug response (10, 20). However, other studies have also reported the association of both alleles with a good clinical response (12). Some studies found the *MTHFR* 677A allele increased chemosensitivity in 5-FU response in colon and breast cancer patients (7). Reduced enzyme activity has also been associated with *MTHFR* 1298C polymorphism (7). In the same way, a study on Bangladeshi patients also found the *MTHFR* polymorphism to be associated with a good clinical 5-FU response (9). Another study found 5-FU sensitivity to be related to the *MTHFR* 1298C allele, with the 1298CC genotype being the most sensitive (11).

The main limitations to this study are the sample size and it being a retrospective study. The sample was small and was only drawn from one hospital in Bangkok, Thailand. Moreover, other non-hematological toxicities such as diarrhea were not considered in this study. Other genes, such as *DPYD* and *TYMS* polymorphisms that can affect toxicity were not studied. Further prospective studies with larger sample sizes need to be carried out to further validate these findings.

## Conclusion

In conclusion, the *MTHFR* 677G>A polymorphism was statistically associated with grade 3-4 hematologic toxicity in both the first and second cycles of treatment of Thai colorectal cancer patients who received 5-FU-based therapy, whereas the *MTHFR* 1298A>C polymorphism had no significant association. The response to 5-FU treatment was not statistically associated with these two single nucleotide polymorphisms. These findings imply that the *MTHFR* 677G>A polymorphism may predict 5-FU toxicity. Therefore, patients receiving 5-FU-based therapy should be aware of their polymorphisms as risk factor for experiencing severe toxicity.

## Data availability statement

The original contributions presented in the study are included in the article/supplementary material. Further inquiries can be directed to the corresponding author.

## Ethics Statement

The studies involving human participants were reviewed and approved by the ethics review committee on Human Research of the Faculty of Medicine Ramathibodi Hospital, Mahidol University, Thailand (MURA2020/1613). The patients/participants provided their written informed consent to participate in this study. Written informed consent was obtained from the individual(s) for the publication of any potentially identifiable images or data included in this article.

## Author contributions

CA and CS designed the research study. PC, ES, TR, SA, and SS collected samples. CA and NV collected clinical data. PJ, PS, MC, AP, and NS performed procedures. CA and NV analyzed data. RL, CA, and NV wrote original draft preparation. CA, NV, and CS contributed to the discussion and reviewed/edited the manuscript. All authors have read and agreed to the published version of the manuscript.

## Funding

This study was supported by grants from the (1) The Office of the Permanent Secretary, Ministry of Higher Education, Science, Research and Innovation No. RGNS 63–196, (2) The Health Systems Research Institute under Genomics Thailand Strategic Fund No. 64-099.

## Conflict of Interest

The authors declare that the research was conducted in the absence of any commercial or financial relationships that could be construed as a potential conflict of interest.

## Publisher’s Note

All claims expressed in this article are solely those of the authors and do not necessarily represent those of their affiliated organizations, or those of the publisher, the editors and the reviewers. Any product that may be evaluated in this article, or claim that may be made by its manufacturer, is not guaranteed or endorsed by the publisher.
